# The influence of substituting corn meal with pelleted winged bean (*Psophocarpus tetragonolobus*) tuber on intake, digestion and rumen fermentation in beef cattle

**DOI:** 10.5713/ab.25.0137

**Published:** 2025-06-24

**Authors:** Pachara Srichompoo, Chaichana Suriyapha, Chanon Suntara, Sompong Chankaew, Metha Wanapat, Anusorn Cherdthong

**Affiliations:** 1 Tropical Feed Resources Research and Development Center (TROFREC), Department of Animal Science, Faculty of Agriculture, Khon Kaen University, Khon Kaen, Thailand; 2 Department of Agronomy, Faculty of Agriculture, Khon Kaen University, Khon Kaen, Thailand

**Keywords:** Alternative Feedstuffs, Energy Source, Pelleting Process, Thai-native Cattle

## Abstract

**Objective:**

The experiment aimed to evaluate the effects of replacing corn meal with winged bean tuber (*Psophocarpus tetragonolobus*) pellets (WBTP) on energy utilization efficiency in Thai indigenous beef cattle.

**Methods:**

A 4×4 Latin square design was used, with WBTP replacing corn meal in the concentrate diet as the independent variable. The four replacement levels were 0%, 33%, 66%, and 100% dry matter.

**Results:**

The intake of nutrients was not affected by replacing cornmeal with WBTP (p>0.05). Substituting WBTP for corn meal did not significantly alter energy utilization (p>0.05). Furthermore, the digestibility of nutrients did not significantly differ across the various WBTP replacement levels. Ruminal pH ranged from 6.73 to 7.04. Blood urea nitrogen values, ranging from 5.50 to 8.75 mg/dL, fell within the typical range for tropical ruminants. No significant differences were observed in total volatile fatty acid concentrations, which ranged from 84.56 to 101.87 mM (p>0.05). Similarly, the proportions of acetate, propionate, and butyrate remained unchanged (p>0.05), despite the addition of WBTP into the concentrate as a replacement for corn meal.

**Conclusion:**

This study highlights the potential of WBTP as a sustainable alternative to corn meal in the diets of Thai-native beef cattle. The study found that replacing corn meal with WBTP at up to 100% had no negative adverse effects on feed intake, nutrient digestibility, ruminal fermentation parameters, or energy efficiency.

## INTRODUCTION

The world’s population is experiencing significant growth, projected to surpass 9 billion by 2050. While the majority of population growth is expected in developing nations, high-income countries are also predicted to see demographic changes, notably a substantial rise in the senior population, resulting in heightened food consumption and production [1]. Consequently, food security and the cost of global food production, particularly for livestock and animal feed, are crucial issues [2,3]. The high price of animal feed, influenced by factors such as transportation costs, climate change, and international conflicts, significantly impacts the cost of animal production [3,4], especially for primary feedstuffs like soybeans (protein source) and corn (energy source) [5–7]. Corn meal serves as the primary energy source extensively used in the preparation of animal feed, leading to increased demand and high prices for corn as animal feed [4,7,8]. In this context, identifying locally available and cost-effective feed alternatives, such as winged bean tuber (*Psophocarpus tetragonolobus*) pellets (WBTP), becomes imperative. Consequently, research has increasingly focused on innovative tuber crops and alternative plant-based feedstuffs that address the dual challenge of cost-efficiency and feed diversity in ruminant diets. These feeds have the potential to enhance local feed diversity, reduce costs, and mitigate feed shortages in specific regions [4,6,7,9].

The winged bean (*Psophocarpus tetragonolobus*, WB), a highly adaptable leguminous plant, is extensively cultivated in tropical regions, including Thailand, for its exceptional nutritional and agronomic benefits. WBs offer numerous benefits, with nearly every part being consumable, including tubers, tender shoots, young leaves, fresh pods, and seeds. Additionally, the bean pods serve as excellent organic fertilizers, enhancing soil structure and increasing nitrogen levels in the soil [10]. Recently, researchers have studied and developed the production of WB tubers which have the potential to be used in animal diet [11,12]. Winged bean tubers (WBT) can yield up to 19.3–22.4 tons/ha/year [12], making them a viable feed source. With approximately 20% crude protein (CP) and 50%–54% non-fiber carbohydrates (NFC), WBT presents a promising alternative to conventional energy feeds in ruminant nutrition [6,12,13]. Given its high starch content, controlling the rate of starch degradation in WBT is crucial for optimizing its use in ruminant diets, particularly to improve starch utilization and maintain stable rumen pH levels. Unnawong et al [6,7] demonstrated that modifying WBT starch through steaming could enhance starch utilization. However, the steaming process has limitations, including the potential loss of nutrients due to prolonged exposure to high temperatures and irregular heat distribution. These factors could potentially reduce the overall nutritional value of the feed.

In contrast, the pelletization process offers a more controlled and consistent method for modifying starch properties. It not only improves the physical characteristics of the feed but also induces beneficial changes in starch through the Maillard reaction and gelatinization, which can further enhance starch utilization in WBT. Srichompoo et al [4] recently created a new WBTP that can be used instead of corn meal. It has good nutritional values and compositions and works well as an energy substitute in concentrate mixtures without affecting the degradability and fermentation *in vitro*. Despite these promising results, there remains a significant research gap, particularly in verifying these findings in animal models such as beef cattle. The investigation findings show that WBTP might function as an alternate energy source to corn meal in beef cattle feed, without impacting feed intake and utilization. The use of WBTP, a locally available resource, not only addresses feed cost issues but also reduces the carbon footprint associated with the transportation and import of conventional feedstuffs like corn meal. This research aimed to investigate the effects of replacing corn meal with WBTP as an alternative energy source in the concentrate diet on rumen fermentation and feed efficiency in Thai-native beef cattle primarily consuming rice straw.

## MATERIALS AND METHODS

### Experiment location

The experiment took place at the Tropical Feed Resources Research and Development Center (TROFREC), which is part of the Department of Animal Science in the Faculty of Agriculture at Khon Kaen University. The university is in Khon Kaen Province, Thailand, at the precise coordinates of 16°26′48.16″N and 102◦49′58.8″E. The experiment was conducted during the winter season, specifically from December 2021 to March 2022, with an average temperature falling between 28°C and 31°C. The research station of the institute was the location where the experimental animals were housed, as it was specifically designed for research purposes. The rumination behavior and digestibility inside the facility were unaffected by the exterior environment. The cattle were accommodated in four enclosures, each measuring 3×5 square meters, and supplied with cement water containers. The animals had comparable age and body weight (BW) before the commencement of the study. The animals were measured, and their initial BW was documented. Subsequently, they were dewormed using Ivomec F from Kos Introtech, and given an injection of vitamin AD3E from Phenix, Anitech Total Solution. Before the commencement of the research, the experimental cattle had a training period of no less than 2 weeks in separate enclosures, allowing the animals to acclimate effectively to the facilities. All experimental cattle remained in good health throughout the study. Following this acclimation period, the animals were subjected to dietary treatments designed to evaluate the effects of WBTP substitution.

### Winged bean tuber pellet preparation

The Faculty of Agriculture, Department of Animal Science, at Khon Kaen University (KKU) in Khon Kaen, Thailand, was the site of the research investigation. The WBT was cultivated and collected at the Faculty of Agriculture, Department of Agronomy, KKU. To achieve a moisture content of less than 10%, the freshly harvested WBT were cut into pieces measuring 3–5 cm and sun-dried for 72 hours. Before being used as a substrate, each WBT was subjected to milling via a 1-mm screen. Subsequently, the WBT powder with a moisture content of 30% was formed into pellets using the procedure described by Seankamsorn et al [14]. The prepared pellets were sun-dried for approximately 72 hours to achieve a moisture content of less than 10%, ensuring optimal storage stability and feed quality.

### Experimental animals, design, and dietary treatments

A quartet of Thai Indigenous beef cattle were used, each with an average BW of 310±20.0 kg. A 4×4 Latin square design was implemented to allocate the cattle. Dietary regimens included four levels of WBTP replacing corn meal, which was initially included at 10% in the control concentrate diet. The corresponding WBTP inclusion levels in the concentrate were 0%, 3.3%, 6.6%, and 10% for the 0%, 33%, 66%, and 100% replacement treatments, respectively. The concentrated diets used in the experiment were produced based on the nutritional needs of Thai native beef cattle, as specified by the recommendation of the Working Committee of Thai Feeding Standard for Ruminants. Each animal was administered a concentrated diet that was equivalent to 1% of their BW two times per day, at 8:00 a.m. and 4:00 p.m. This was followed by the unrestricted feeding of rice straw. Table 1 presents the chemical compositions of the dietary concentrate combination treatment, rice straw, corn meal, and WBTP. The concentrate diets were created using local feed ingredients that had comparable CP levels, ranging from 14.87% to 15.71% CP, and similar gross energy content, ranging from 4.06 to 4.02 megacalories/kg DM, in each treatment. Each session of the research lasted 21 days, and it was conducted over four consecutive periods. The initial 14-day period was intended to facilitate the subjects’ adaptation to the diet provided. The subsequent 7-day period was dedicated to the collection of samples using the total collection technique in specialized enclosures that were specifically designed to measure the digestibility of fecal matter. The quantity of food ingested and rejected was documented daily. At the commencement and conclusion of each interval, black-and-white images were recorded.

### Chemical analysis and sample retrieval

The cattle were relocated to the metabolism enclosures to investigate digestibility after the trial concluded. Consecutive samples of concentrated diet, WBTP, rice straw, and leftover food were obtained and preserved at a temperature of −20°C until further investigation. Fecal samples were collected, measured, documented, and combined by the animals during the final seven days of each session. Finally, the samples were maintained at a temperature of −20°C. After a 7-day collection period, defrosted samples from each animal were pooled and analyzed. The portions were subsequently thoroughly mingled, and smaller portions of the feed that was consumed and the feed that was not consumed were taken. Feed and fecal samples were dried in a forced-air oven at 60°C for 72 hours. After drying, they were ground using a Cyclotec Mill (Tecator) to achieve a particle size capable of passing through a 1 mm screen.

The standard method of the Association of Official Analytical Chemists [15] was used to analyze the chemical compositions of feed and feces. This included the constituents of DM (no. 967.03), organic matter (OM), and ash (no. 492.05), as well as ether extract (EE, no. 920.39). A nitrogen analyzer (Leco FP828 Nitrogen Analyzer; LECO) was employed to evaluate the CP content. The following procedure was used to compute NFC: According to Holtshausen [16]:


(1) NFC (%)=100-(CP [%]+EE [%]+NDF [%]+ash [%])

The neutral detergent fiber (NDF) and acid detergent fiber (ADF) contents of the samples were determined by simmering them in NDF and ADF solutions. Additionally, the analysis was conducted in accordance with the methodology established by Van Soest et al [17], and α-amylase was added. The gross energy (GE) content of experimental diets was determined by employing an adiabatic calorimeter bomb (AC500, LECO). The procedure of Keawpila et al [18] was used to determine the estimated metabolizable energy (ME) content of experimental diets.


(2) ME=0.1913OM+0.0956EE-0.0992ADF-6.1887

Hematological specimens were obtained from the vein located in the jugular on the final day of each phase at two specific time points: immediately before the morning meal and 4 hours after the beginning of nourishment. Concurrently with the collection of rumen fluid, hematological samples have been taken from the jugular vein., with an estimated volume of 10 mL. Each tube contained 12 mg of ethylenediaminetetraacetic acid to prevent coagulation. To separate the plasma, it was centrifuged at a force that was 500 times the acceleration due to gravity for 10 minutes. The material was thereafter maintained at −20°C until analysis of blood urea nitrogen (BUN) concentrations was conducted using the L-type Wako UN diagnostic device manufactured in Tokyo, Japan. The diacetyl monoxime method was subsequently employed to implement an automated colorimetric technique. Using a gastric tube connected to a vacuum compressor, ruminal fluid samples (100 mL) were collected on the final day of the feeding study, both before and four hours after feeding. The early part of the rumen fluid was eliminated to prevent saliva contamination. The pH of the ruminal fluid was swiftly measured using a HANNA Instruments HI 8424 microcomputer from Singapore. The fluid samples were segregated into two separate parts after the pH measurements. The initial 45 mL of fluid samples were transferred to a 60 mL plastic container and combined with 9 mL of 1 M H_2_SO_4_. This study aimed to ascertain the amounts of volatile fatty acids (VFA), namely acetic acids (C2), propionic acids (C3), and butyric acids (C4). The second sample of ruminal fluid, measuring one milliliter and containing nine milliliters of 10% formaldehyde in formalin, was designated for assessing bacterial and protozoal populations. The ruminal fluid samples were centrifuged for 15 minutes at a force of 16,000×g after collection. The supernatant, the liquid layer above the debris, was then tested to quantify the VFA present. VFA concentration was determined using gas chromatography (Nexis GC-2030; Shimadzu) with a flame ionization detector and a capillary column (DB-WAX 30 m, 0.25 mm, 0.25 μm; Agilent Technologies). The bacterial and protozoal populations were quantified using hemacytometers (Tiefe depth 0.1 mm and 0.0025 mm^2^; ISO LAB Laborgerate) and a microscope, under the technique outlined by Galyean et al [19]. The calculation of apparent digestibility and gross energy intake (GEI) was done according to the report of Suriyapha et al [5] following:


(3) Apparent digestibility (%)=(Nutrient intake-Nutrient in feces)×100/Nutrient intake

(4) GEI (Mcal/day)=DM intake of diet (kg/day)×GE content in diet (Mcal/kg DM)

(5) MEI (Mcal/day)=DM intake of diet (kg/day)×ME content in diet (Mcal/kg DM)

(6) MEI:GEI=MEI/GEI

Where: GEI = gross energy intake, GE = gross energy, ME = metabolizable energy, MEI = metabolizable energy intake, MEI:GEI = energy efficiency (MEI to GEI ratio).

### Statistical analysis

The SAS Proc GLM procedure was employed to evaluate the data as a 4×4 Latin square design, as indicated by the following equation [20]:


(7) $${\text{Y}}_{\text{ijk}}=\mu +{\text{M}}_{\text{i}}+{\text{A}}_{\text{iij}}+{\text{P}}_{\text{k}}+{ɛ}_{\text{ijk}}$$

Where Y_ijk_, is the observation from animal j, receiving diet i, in period k; μ is the overall mean, M_i_ is the effect of the level of WBTP (i = 1, 2, 3, 4), Aj is the effect of animal (j = 1, 2, 3, 4); P_k_ is the effect of the period (k = 1, 2, 3, 4); and ɛijk is the residual effect. To identify any significant differences among the treatments, Tukey’s Multiple Comparison Test was employed. Orthogonal polynomials were utilized to statistically compare the treatment trends.

Ruminal VFA concentrations and proportions, along with BUN levels, were analyzed using a repeated-measures mixed model that included baseline values (0 h pre-feeding) as covariates for the initial values at time 0 h before feeding using the Proc Mixed procedure of SAS [20] as shown in the model.


(8) $${\text{Y}}_{\text{ijkm}}=\mu +{\text{P}}_{\text{i}}+{\text{G}}_{\text{k}}+{\text{t}}_{\text{m}}+{\text{T}}_{\text{j}}+{ɛ}_{\text{ijkm}}$$

Where Y_ijkm_ is the observation for a specific variable, μ is the overall mean, P_i_ is the random effect of period, G_k_ is the random effect of cow, tm is the random effect of sampling time (0 h pre- and 4 h post-feeding), and T_j_ is the fixed effect of treatment. The residual random term is denoted by ɛ. The trend effect of WBTP substitution levels was investigated using orthogonal polynomials.

## RESULTS

### Feed intake

Table 2 illustrates the impact of substituting corn meal in the diet with WBTP on feed intake. Feed intake across all WBTP replacement levels showed no statistically significant differences (p>0.05), indicating the potential of WBTP as a viable substitute for corn meal without compromising intake.

### Nutrient intake, energy utilization, and digestibility

The effects of substituting corn meal with WBTP on nutrient intake, energy utilization, and digestibility are presented in Table 3. The intake of OM, DM, EE, NDF, ADF, and NFE was not affected by replacing corn meal with WBTP (p>0.05). Substituting WBTP for corn meal did not significantly alter GEI, MEI, or their ratio (p>0.05). Furthermore, the digestibility of DM, OM, CP, EE, NDF, ADF, and NFE did not significantly differ between the various WBTP replacement levels (p>0.05).

### Ruminal pH, blood urea nitrogen, and microbial population

The effects of substituting corn meal with WBTP on ruminal pH, BUN, and microbial population are presented in Table 4. Ruminal pH ranged from 6.73 to 7.04. BUN values, ranging from 5.50 to 8.75 mg/dL, fell within the typical range for tropical ruminants. In the present study, at 0 hours post-feeding, protozoal populations increased significantly with 100% WBTP substitution.

### Ruminal volatile fatty acids

Table 5 presents the effects of replacing corn meal with WBTP on total VFA concentrations and VFA profiles in the rumen of Thai-native beef cattle. No significant differences were found in total VFA concentrations ranging from 84.56 to 101.87 mM (p>0.05). Similarly, the proportions of C2, C3, and C4 remained unchanged (p>0.05), despite the incorporation of WBTP into the concentrate as a replacement for corn meal.

## DISCUSSION

### Feed intake

The findings of this study suggest that the total DM intake (DMI) of Thai-native cattle fed a rice straw-based diet is within the typical range of 2.39% to 2.69% of BW [5,7]. Furthermore, replacing corn meal in the concentrate diet with WBTP did not negatively impact total DMI and nutrient intake compared to the control group. Pelleting, which involves heat treatment, can disrupt cellular structures, alter starch–protein interactions, and induce starch gelatinization, thereby enhancing the palatability of WBTP in terms of taste, odor, and aroma [6,21]. Additionally, the pelleting process, which improves nutrient availability in WBTP, may have contributed to the comparable digestibility observed between the 100% WBTP and control groups. Moreover, substituting corn meal with WBTP in the concentrate diet can balance feed utilization by improving the sensory qualities of the feed [21]. Building on the feed intake analysis, the following section explores the impact of WBTP substitution on nutrient utilization and energy balance. With feed intake remaining unaffected, the study further evaluates nutrient digestibility and energy utilization under WBTP-based diets.

### Nutrient intake, energy utilization, and digestibility

Digestibility of DM, OM, CP, EE, NDF, and ADF was unaffected by WBTP substitution at levels up to 100%, potentially improving overall health and productivity. Starch gelatinization during processing increases the surface area for microbial activity, which may maintain digestibility at levels similar to corn meal [22]. The rate of carbohydrate fermentation is influenced by several factors, including monosaccharide type and arrangement, molecular size, and feed physical structure [23].

Replacing corn meal with WBTP sustained energy intake levels in Thai-native beef cattle, demonstrating its efficacy in meeting the energy requirements of ruminants without adverse effects. The recommended metabolizable energy for maintenance (MEm) in Thai-native cattle is 0.12 Mcal/kg BW^0.75^/day. In this experiment, the MEI varied between 0.20 and 0.22 Mcal/kg BW^0.75^/day, indicating a favorable energy balance sufficient to sustain growth. Replacing corn meal with WBTP maintained both GEI and MEI, likely due to the consistent DMI, which ensured sufficient overall energy intake for growth. In contrast, Unnawong et al [7] reported that feeding Thai-native beef cattle a concentrated diet containing modified WBTP increased energy intake, possibly due to the appetite-stimulating effects of modified starch. In the present study, the MEI/GEI efficiency (0.60–0.63) was consistent with values reported by Kongphitee et al [24], who observed an MEI/GEI ratio of 0.56–0.70 when using cassava pulp in the diets of Thai-native beef cattle. Therefore, the energy requirements of Thai-native beef cattle can be met by substituting WBTP for corn meal in the concentrate diet. While WBTP substitution showed no adverse effects on energy utilization, long-term studies are needed to assess potential impacts on growth performance and production efficiency.

### Ruminal pH, blood urea nitrogen, and microbial population

The ruminal pH is within the ideal range for microbial activity (pH 6.20 to 7.00) [25]. This pH range is crucial for effective feed degradation and rumen health. Agarwal et al [26] also noted that ruminal microbes are most active within a pH range of 6.0 to 7.0, depending on rumen conditions. The variation in ruminal pH at 0 hours post-feeding may be due to inconsistent feeding behavior under ad libitum conditions [27,28]. Although ruminal pH fluctuations were within the optimal range, the slight variability observed immediately post-feeding suggests that WBTP may influence the buffering capacity of ruminal contents, possibly due to changes in starch composition and fermentation dynamics. On the other hand, the stable ruminal pH observed after four hours of feeding and throughout the day suggests that the rumen microbial population adapted to the diet over time [4,29]. Even at WBTP inclusion levels as high as 100%, ruminal fermentation and pH remained stable, demonstrating resilient ruminal metabolism and consistent microbial activity [25,26].

BUN concentrations in Thai-native cattle typically range from 6.30 to 25.50 mg/dL [5,7]. In this study, BUN levels were unaffected by WBTP substitution, likely due to the similar CP and nitrogen sources in the feed. Unnawong et al [7] also reported no significant changes in BUN and ruminal NH_3_-N levels when using steamed WBT in ruminant feed, supporting the comparable BUN levels observed here.

Before feeding, rumen protozoal populations may fluctuate due to factors such as circadian rhythms, nutrient availability, and microbial interactions. Protozoa feed on bacteria and starch, and in the absence of fresh feed, they adjust their population based on the availability of fermentable substrates [19]. Moreover, Dennis [30] found that rumen protozoa populations increased with higher energy and nitrogen levels, such as those in diets containing pelleted mixtures. Pelleted feed enhances the fermentation environment, supporting protozoal growth. Galyean [19] suggested that the availability of glucose, a key nutrient for protozoa, may be influenced by the physical properties of pellets. However, protozoal populations did not change after four hours of feeding, likely indicating that the protozoal community had adapted to the diet during the 21-day feeding period.

### Ruminal volatile fatty acids

The VFA profiles and total VFA concentrations remained stable four hours post-feeding, likely due to the similar nutrient composition of WBTP and corn meal [4], resulting in comparable ruminal fermentation dynamics [31]. The consistent VFA concentrations across treatments highlight the ability of WBTP to maintain stable fermentation patterns. Although not statistically significant, the slight increase in propionate at 66% WBTP substitution may suggest improved glucogenic potential, meriting further investigation. Srichompoo et al [4] also found that substituting WBTP for corn meal did not alter VFA profiles during *in vitro* fermentation after four hours of incubation. The heat produced during pelleting or steaming may have modified WBTP’s amylopectin, creating branched, gelatinized structures [7]. These changes produce denser starch granules, often referred to as resistant starch, which reduces starch solubility in the rumen [21] Unnawong et al [7] demonstrated that increasing the level of steamed WBT up to 30% in the diet resulted in similar ruminal VFA concentrations and profiles compared to the control group.

## CONCLUSION

This study highlights the potential of WBTP as a sustainable alternative to corn meal in the diets of Thai-native beef cattle. By replacing corn meal with WBTP at levels up to 100%, the study demonstrated no adverse effects on feed intake, nutrient digestibility, ruminal fermentation parameters, or energy efficiency. These findings align with the objective of identifying locally available feed resources that maintain optimal nutritional performance in ruminants. Future studies should evaluate the long-term impacts of WBTP substitution on production efficiency, including growth performance, carcass quality, and economic returns. Additionally, exploring the scalability of WBTP use across diverse ruminant production systems is essential to validate its practical application.

## Figures and Tables

**Table 1:** Components and chemical formulations of the experimental diet

Ingredients (% DM)	Replacement level				Corn meal	WBTP	RS

	Control	33%	66%	100%			
Cassava chip^1)^	40.00	40.00	40.00	40.00			
Corn meal	10.00	6.66	3.33	0.00			
Rice bran	14.00	14.00	14.00	14.00			
WBTP	0.00	3.33	6.66	10.00			
Soybean meal	14.00	14.00	14.00	14.00			
Palm kernel	16.00	16.10	16.20	16.30			
Molasses	3.00	3.00	3.00	3.00			
Salt	1.00	0.90	0.80	0.70			
Urea	1.00	1.00	1.00	1.00			
Minerals and vitamins^2)^	1.00	1.00	1.00	1.00			
Chemical composition							
Dry matter (DM) %	89.99	89.49	89.43	88.78	87.51	95.21	94.21
Organic matter (% DM)	93.68	93.91	93.93	93.98	96.24	94.52	86.23
Crude protein (% DM)	14.87	15.14	15.43	15.71	7.94	15.72	2.43
Ether extract (% DM)	4.81	4.74	4.36	4.22	3.34	0.62	0.91
Neutral detergent fiber (% DM)	20.55	21.14	23.72	25.56	22.13	24.74	64.32
Acid detergent fiber (% DM)	14.21	14.68	14.68	14.92	8.32	9.41	40.24
Acid detergent lignin (% DM)	1.61	1.68	1.47	1.39	1.53	0.73	7.92
Non-fiber carbohydrate	53.35	52.89	50.42	48.49	66.59	58.92	32.34
Cellulose (% DM)^3)^	12.60	13.00	13.21	13.53	6.79	8.68	32.32
Hemicellulose (% DM)^2)^	6.34	6.46	9.04	10.64	13.81	15.33	24.08
Gross energy (Mcal/kg DM)	4.06	4.05	4.04	4.02	3.41	1.24	3.82
Metabolizable energy (Mcal/kg DM)	2.58	2.57	2.57	2.56	2.80	2.63	1.89

1) Cassava chip contained: 90.17% dry matter, 2.04% pure protein, 0.77% ether extract, 27.5% neutral-detergent fiber, 4.01% acid-detergent fiber, and 66.74% non-fiber carbohydrate. Four minerals and micronutrients (per kilogram): Vitamin A contains 10,000,000 IU, Vitamin E contains 70,000 IU, and Vitamin D contains 1,600,000 IU. Fe is 50 g, Zn is 40 g, Mn is 40 g, Co is 0.1 g, Cu is 10 g, Se is 0.1 g, and I is 0.5 g.

2) Hemicellulose was determined as NDF–ADF.

3) Cellulose was determined as ADF–ADL.

WBTP, winged bean tubers pellet; RS, rice straw.

**Table 2:** Impact of replacing corn meal with winged bean tuber pellets (WBTP) on feed consumption (DM basis) in Thai native steers

Items	Replacement level				p-value	SEM	Contrast	
	
	Control	33%	66%	100%			Linear	Quadratic
Concentrate intake								
kg/day	2.42	2.38	2.40	2.35	1.00	0.17	1.00	0.99
% BW	0.98	0.99	0.99	0.99	0.72	0.67	0.66	0.79
g/kg BW^0.75^	34.94	31.65	35.71	31.17	2.40	1.20	0.52	0.80
Rice straw intake								
kg/day	4.35	4.42	4.61	4.64	0.61	0.18	0.67	0.93
% BW	1.54	1.40	1.70	1.48	0.24	0.10	0.31	0.71
g/kg BW^0.75^	63.08	58.97	68.88	62.04	0.32	3.65	0.40	0.74
Total intake								
kg/day	6.77	6.79	7.00	6.99	0.68	0.23	0.74	0.95
% BW	2.52	2.39	2.69	2.47	0.24	0.10	0.31	0.71
g/kg BW^0.75^	98.02	90.62	104.59	93.20	0.18	4.46	0.27	0.69

SEM, standard error of the means.

**Table 3:** Impact of replacing corn meal with winged bean tuber pellets (WBTP) on nutritional consumption and digestibility in Thai native steers

Items	Replacement level				p-value	SEM	Contrast	
	
	Control	33%	66%	100%			Linear	Quadratic
Nutrient intake (kg/day)								
Organic matter	6.27	6.30	6.49	6.49	0.72	0.174	0.77	0.93
Crude protein	0.50	0.51	0.53	0.53	0.77	0.024	0.82	0.96
Ether extract	0.17	0.17	0.16	0.16	0.86	0.007	0.89	0.88
Neutral detergent fiber	3.34	3.40	3.60	3.67	0.21	0.120	0.29	0.96
Acid detergent fiber	2.13	2.17	2.25	2.27	0.51	0.075	0.58	0.88
Non-fiber carbohydrate	2.82	2.84	2.84	2.81	0.99	0.094	0.99	0.81
Estimate energy intake								
GE (Mcal/day)	27.35	27.62	28.43	28.53	0.73	4.456	0.79	0.93
GE (Mcal/kg BW^0.75^)	0.40	0.37	0.43	0.38	0.19	0.018	0.29	0.67
ME (Mcal/day)	15.05	15.17	15.60	15.65	0.78	4.456	0.83	0.95
ME (Mcal/kg BW^0.75^)	0.22	0.20	0.23	0.21	0.17	0.009	0.27	0.65
ME:GE	0.55	0.55	0.55	0.55	0.92	0.002	0.92	0.70
Nutrient digestibility (%)								
Dry matter	75.55	75.14	75.12	71.26	0.54	2.330	0.59	0.50
Organic matter	78.98	79.03	74.99	78.16	0.43	1.917	0.47	0.45
Crude protein	74.93	73.76	71.17	77.30	0.65	3.395	0.51	0.22
Ether extract	85.48	86.10	85.36	83.32	0.61	1.511	0.61	0.40
Neutral detergent fiber	62.92	63.59	65.63	65.60	0.61	1.752	0.48	0.82
Acid detergent fiber	57.92	59.39	59.70	61.46	0.80	2.481	0.68	0.95
Non-fiber carbohydrate	96.14	95.98	95.14	95.26	0.96	1.589	0.83	0.88

SEM, standard error of the means; GE, gross energy; ME, metabolizable energy.

**Table 4:** Impact of replacing corn meal with winged bean tuber pellets (WBTP) on ruminal pH, blood urea nitrogen, and microbial population in Thai native steers

Items	Replacement level				p-value	SEM	Contrast	
	
	Control	33%	66%	100%			Linear	Quadratic
Ruminal pH								
0 h	6.99	6.88	6.94	6.98	0.67	0.07	0.37	0.14
4 h	6.75	6.72	6.80	6.87	0.66	0.09	0.27	0.34
Mean	6.88	6.81	6.87	6.93	0.67	0.07	0.28	0.16
Blood urea nitrogen (mg/dL)								
0 h	6.75	5.50	6.75	6.25	0.85	1.15	0.81	0.73
4 h	8.50	6.50	8.25	7.25	0.53	1.06	0.43	0.60
Mean	7.63	6.00	7.50	6.75	0.68	1.05	0.58	0.64
Bacterial number (×10^10^ cell/mL)								
0 h	8.34	8.66	8.62	8.34	0.68	1.05	0.44	0.12
4 h	8.94	8.35	8.37	8.53	0.16	0.16	0.22	0.10
Mean	8.64	8.51	8.49	8.44	0.76	0.15	0.63	0.75
Protozoal number (×10^8^ cell/mL)								
0 h	0.82^c^	1.22^b^	1.22^b^	1.42^a^	0.02	0.05	0.04	0.44
4 h	1.25	1.35	1.00	1.25	0.61	0.09	0.66	0.69
Mean	1.04	1.29	1.11	1.34	0.22	0.07	0.56	1.00

a–c Means with different letters in a role are significantly different at p<0.05.

SEM, standard error of the means.

**Table 5:** Impact of replacing corn meal with winged bean tuber pellets (WBTP) on volatile fatty acid profiles in Thai native steers

Items	Replacement corn meal WBTP level						Contrast	

	Control	33%	66%	100%	p-value	SEM	Linear	Quadratic
Total volatile fatty acids (mmol)								
0 h	95.39	99.74	78.76	91.44	0.26	14.58	0.86	0.28
4 h	99.42	104.01	90.36	97.64	0.86	18.74	0.24	0.53
Mean	97.41	101.87	84.56	94.54	0.60	7.29	0.89	0.33
Acetic acid (mol/100 mol)								
0 h	65.15	69.40	61.16	66.82	0.44	4.11	0.87	0.39
4 h	65.12	65.84	63.41	66.21	0.91	2.46	0.47	0.63
Mean	65.14	67.62	62.29	66.52	0.82	2.54	0.43	0.24
Propionic acid (mol/100 mol)								
0 h	23.93	22.04	27.28	23.04	0.97	2.34	0.57	0.46
4 h	24.80	24.63	26.19	22.68	0.86	1.52	0.29	0.44
Mean	24.37	23.34	26.74	22.84	0.99	7.44	0.34	0.45
Butyric acid (mol/100 mol)								
0 h	10.92	8.56	11.56	10.14	0.67	0.72	0.84	0.15
4 h	10.08	9.53	10.40	11.11	0.92	0.87	0.53	0.14
Mean	10.50	9.04	10.98	10.62	0.74	0.77	0.39	0.70

SEM, standard error of the means.
